# Construction and analysis of a plant non-specific lipid transfer protein database (nsLTPDB)

**DOI:** 10.1186/1471-2164-13-S1-S9

**Published:** 2012-01-17

**Authors:** Nai-Jyuan Wang, Chi-Ching Lee, Chao-Sheng Cheng, Wei-Cheng Lo, Ya-Fen Yang, Ming-Nan Chen, Ping-Chiang Lyu

**Affiliations:** 1 Institute of Bioinformatics and Structural Biology, National Tsing Hua University, Hsinchu, Taiwan; 2 Department of Computer Science, National Tsing Hua University, Hsinchu, Taiwan; 3 Institute of Bioinformatics and Systems Biology, National Chiao Tung University, Hsinchu, Taiwan; 4 Department of Medical Science, National Tsing Hua University, Hsinchu, Taiwan; 5 Graduate Institute of Molecular Systems Biomedicine, China Medical University, Taichung, Taiwan

## Abstract

**Background:**

Plant non-specific lipid transfer proteins (nsLTPs) are small and basic proteins. Recently, nsLTPs have been reported involved in many physiological functions such as mediating phospholipid transfer, participating in plant defence activity against bacterial and fungal pathogens, and enhancing cell wall extension in tobacco. However, the lipid transfer mechanism of nsLTPs is still unclear, and comprehensive information of nsLTPs is difficult to obtain.

**Methods:**

In this study, we identified 595 nsLTPs from 121 different species and constructed an nsLTPs database -- nsLTPDB -- which comprises the sequence information, structures, relevant literatures, and biological data of all plant nsLTPs http://nsltpdb.life.nthu.edu.tw/.

**Results:**

Meanwhile, bioinformatics and statistics methods were implemented to develop a classification method for nsLTPs based on the patterns of the eight highly-conserved cysteine residues, and to suggest strict Prosite-styled patterns for Type I and Type II nsLTPs. The pattern of Type I is C X_2 _V X_5-7 _C [V, L, I] × Y [L, A, V] X_8-13 _CC × G X_12 _D × [Q, K, R] X_2 _CXC X_16-21 _P X_2 _C X_13-15_C, and that of Type II is C X_4 _L X_2 _C X_9-11 _P [S, T] X_2 _CC X_5 _Q X_2-4 _C[L, F]C X_2 _[A, L, I] × [D, N] P X_10-12 _[K, R] X_4-5 _C X_3-4 _P X_0-2 _C. Moreover, we referred the Prosite-styled patterns to the experimental mutagenesis data that previously established by our group, and found that the residues with higher conservation played an important role in the structural stability or lipid binding ability of nsLTPs.

**Conclusions:**

Taken together, this research has suggested potential residues that might be essential to modulate the structural and functional properties of plant nsLTPs. Finally, we proposed some biologically important sites of the nsLTPs, which are described by using a new Prosite-styled pattern that we defined.

## Background

Lipids are hydrocarbons insoluble in water but soluble in organic solvents. They are commonly translocated among subcellular membranes to enable various metabolic activities [1]. Lipid transfer proteins (LTPs) have been found in animals, plants and some fungi, and they exist in many tissues with various sizes and functions [1-3]. LTPs play an important role not only in plant but also in human. It mediates *in vitro *the transfer of all common phospholipids, cholesterol and gangliosides between membranes [4,5]. The term plant "nonspecific lipid transfer proteins" indicates that LTPs can associate with various phospholipids with broad specificity [6]. The first known plant lipid transfer protein was isolated from potato tuber in 1975 by Kadar [7]. At present, much more nsLTPs have been found in monocots, dicots and gymnosperms, etc [3]. Plant nsLTPs are a kind of small (usually 6.5 to 10.5 kDa), basic (isoelectric point, or pI, usually falls between 8.5 and 12) and stable (with four conserved disulfide bonds) proteins. They can be isolated from various plants, *e.g*., Arabidopsis, rice, barely, wheat, maize, caster bean, and spinach leaf [8-15]. All the identified nsLTPs show high homology in protein sequence and share similar characteristics. NsLTPs are stabilized by eight conserved cysteine residues forming four disulfide bonds and they usually contain signal peptides in the N-terminus [1]. Previous studies showed that nsLTPs can be divided into two main groups according to their molecular weight: nsLTP1 (9 kDa) and nsLTP2 (7 kDa) [16]. These two groups exhibit different disulfide bond patterns. The disulfide bond linkages of nsLTP1 at Cys_1_-Cys_6 _and Cys_5_-Cys_8 _differ from those of nsLTP2 at Cys_1_-Cys_5 _and Cys_6_-Cys_8_. The major difference is observed at the C_6_-X-C_8 _motif. For the CXC motif in nsLTP1, × is a hydrophilic residue, for example asparagine; however, in nsLTP2, a hydrophobic residue, such as leucine or phenylalanine, was found at the × position. These conserved hydrophilic or hydrophobic residues may play important roles in the biological functions of nsLTPs [17].

Several plant nsLTP structures have been determined. Three dimensional structures of either ligand-free and ligand-bound forms of nsLTPs are available [9,10,12,18]. The structure of nsLTP1 is composed of four alpha helices and a flexible stretching C-terminus [19,20]. The four alpha helices are connected by flexible loops and stabilized by the four disulfide bonds [10,12,18]. A typical characteristic of nsLTPs is the existence of an internal hydrophobic cavity running through the molecule. The cavity allows the binding of one or two monoacyl lipids, diacylated lipids, or some hydrophobic molecules [18,21]. The hydrophobic cavity in nsLTP1 shows a tunnel-like conformation, and nsLTP2 exhibits a triangular conformation [19]. The major structural difference between nsLTP1 and nsLTP2 is the size of the hydrophobic cavity; the cavity of an nsLTP1 is usually larger than that of an nsLTP2 protein [10,18,22]. In recent years, increasing studies have reported that plant nsLTPs are involved in many crucial biological functions but the mechanisms responsible for these functions are unclear yet.

Several biological functions of plant nsLTPs have been identified, inclusive of mediating phospholipid transfer, involving in plant defence activity against bacterial and fungal pathogens, and participating in the assembly of hydrophobic protective layers of surface polymers such as the formation of cutin [23,24]. NsLTPs were found accumulated at the surface of certain tissues at a high concentration [25], which may be correlated with the adaptation to different environmental stresses [26]. Several studies pointed out that the expression of nsLTPs can be induced by environmental stresses like extreme temperatures, osmosis pressures and drought [27]. Furthermore, nsLTPs exhibit defence activities toward blight or pathogens because of their high thermal stability and resistance to proteases [23,28]. In addition, nsLTPs are involved in the formation of beer foam [29] and in food allergy to processed fruits [30]. Jeroen Nieuwland et al. postulated that nsLTPs can associate with hydrophobic cell wall compounds and disrupt the cell wall or facilitate the extension of cell wall [31]. These features of nsLTPs suggest that their functions are very diverse, and these features may exist because of their ability to bind and/or carry hydrophobic molecules such as fatty acid or fatty acid derivatives [25].

There is no golden standard for the identification and classification of nsLTPs because of their unclear lipid transfer mechanisms and the insufficiency of publicly available data. In the last twenty years, nsLTPs were mainly categorized into two subfamilies based on their molecular weights, nsLTP1 (~9 kDa) and nsLTP2 (~7 kDa) [3]. Nevertheless, this method is inadequate for categorizing many newly identified nsLTPs [7]. In 2008, Boutrot et al. proposed a new classification for nsLTPs using the putative mature form of rice, wheat, and Arabidopsis thaliana. The authors divided nsLTPs into nine types (from I to IX) according to their sequence similarities (see Additional File 1) [32]. Some recent papers minorly modified this classification system using a very limited number of sequences (see Additional File 2) [33,34].

Plant non-specific lipid transfer proteins are one of the most well-known proteins that are widely distributed in the plant kingdom. Our wet-lab laboratory has been studying nsLTPs for years, but there is still much unknown space left about these sequence highly-diverse proteins. Importantly, there is no nsLTPs database systematically collecting and organizing relevant data about nsLTPs. Boutrot et al. had identified and classified 267 nsLTPs sequences in 2008 [32], but their method still failed to classify many nsLTPs (see Table 1) [32]. This works aimed to establish an nsLTPs database, develop a robust classification method for nsLTPs and formulate Prosite signature patterns for the identification of nsLTPs as well as the key residues for the structural stability or the lipid binding ability of nsLTPs.

**Table 1:** Comparison of several published nsLTP datasets

Authors [reference number]	Year of publication	Species	Total number of nsLTP sequences	Number of non-redundant nsLTP sequences
Gautier, *et al.* [32]	2008	3	251	131
Liu, *et al.* [33]	2010	1	135	75
Edstam, *et al. *[52]	2011	5	149	81
This study	2011	121	1,395	595

## Methods

### Databases and web-based tools utilized

#### NCBI http://www.ncbi.nlm.nih.gov/

The National Center for Biotechnology Information provides many public databases and tools relating to biotechnology. First, we established a non-redundant protein sequence dataset by retrieving data from NCBI RefSeq and Genbank; then, we used BLAST [35] to search for homologous sequences for each sequence in our dataset.

#### ExPASY http://expasy.org/

The ExPASy (Expert Protein Analysis System) database is established by Swiss Institute of Bioinformatics (SIB) and European Bioinformatics Institute (EBI), such as Swiss-Prot, UniProtKB and TrEMBL. Swiss-Prot and TrEMBL provide many information relating to protein sequence, structure and function (e.g., domains structure, post-translational modifications, variants) [36].

#### TARGETP http://www.cbs.dtu.dk/services/TargetP

The TargetP 1.1 Server is a web based tool to predict the subcellular location of eukaryotic proteins [37].

#### SignalP 3.0 Server http://www.cbs.dtu.dk/services/SignalP/

The SignalP 3.0 Server is a sequence prediction server that allows user to submit the sequence query and receive the result about presence and location of signal peptide cleavage sites in amino acid sequences from different organisms. In this study, all identified nsLTPs were analyzed for presence of potential signal peptide cleavage sites by using this tool. After removing the signal peptide of all nsTPs in our database, we got putative mature-form nsLTPs sequences. Each putative mature nsLTP sequence was validated through the analysis of the 8-cysteine residue motif (8-Cys motif):

Cys_1_-Xn-Cys_2_-Xn-Cys_3_Cys_4_-Xn-Cys_5_XCys_6_-Xn-Cys_7_-Xn-Cys_8_

After removing proteins improbable to be nsLTPs, we identified 1,395 putative nsLTP sequences. Then we constructed a database and a web-based user interface collecting all these putative nsLTPs and relevant information. Additionally, in order to make our results more reliable, we deleted any redundant sequences with 100% sequence identities and finally got 595 putative nsLTP sequences; these sequences were employed for subsequent protein analysis and evolutionary study.

#### Protein Data Bank http://www.pdb.org/

The protein structure files utilized in this work were obtained from the Protein Data Bank (PDB) [38].

#### Prosite database ftp://ftp.expasy.org/databases/prosite/

The Prosite database is a collection of annotated motif descriptors from protein families and domains [39-42]. These descriptors, or patterns, are extracted from SWISS-PROT protein databases. Each pattern is recorded with two files: PROSITE.DAT is a computer readable text file providing all information necessary to programs that will scan sequences with patterns and/or matrices, and PROSITE.doc contains textual information and the documentation of patterns listed in PROSITE.DAT.

The version of Prosite we used was 20.8. After careful tests, we found that, although this version of Prosite possess 1,331 patterns, few of them are related with nsLTPs and most mature nsLTP sequences could not be recognized by those patterns. In this study, we examined the eight well-conserved cysteine region of collected nsLTPs and finally proposed new Prosite-styled patterns for Type I and Type II nsLTPs.

### Data mining and Hidden Markov model

The standalone BLAST (version 2.2.17) [33] was utilized as the search engine, by using which we searched all known plant nsLTP sequences against the SwissProt protein sequence database. For every known nsLTP, homologous sequences from plant organisms with sequence identities >15% were considered as candidate nsLTP sequences. Then, candidates without 8-Cys motif were filtered out. After further removing redundant homologous sequences with 100% sequence identities, we manually examined every remaining candidate nsLTP sequence and thus identified 595 nsLTPs.

### Sequence alignment and phylogenetic tree reconstruction

To examine the phylogenetic relationships of the nsLTPs identified in this study, we used ClustalW (version 2.0.12), a well-known multiple sequence alignment method, to obtain all the pairwise sequence similarities between nsLTP sequences. After refining the alignment results manually, we utilized the PHYLIP package v3.67 [43] to construct the phylogenetic tree of nsLTPs by using the UPGMA (Unweighted Pair Group Method with Arithmetic Mean) [44] and the neighbor-joining [45] clustering methods. Finally, MEGA4 [46] and Dendroscope [47] software packages were recruited to draw the tree graphs. In the web interface of our database, BioEdit (v7.0.9.0) was also utilized to compute amino acid identities and visualize sequence alignments.

## Results and discussion

### Classification based on sequence similarities

In this study, we characterized 595 nsLTPs. The presence of signal peptide for each protein was predicted by using the SignalP 3.0 program, and we found that 98% of the nsLTP precursors were initially synthesized with a signal peptide of 7-49 amino acids. The main characteristic of plant nsLTPs was the presence of eight cysteine residues at highly conserved positions, the spanning of which forms a common sequence pattern:

C-Xn-C-Xn-CC-Xn-CXC-Xn-CXn-C (8-Cys motif).

This 8-Cys motif is consensus in nsLTPs, but it could not be used to classify nsLTPs. To classify nsLTPs, we modified Boutrot's nine-type classification into a five-type system (see Table 2 and Table 3). After analyzing the classified nsLTPs, we found that (1) Types I and II are shared by all the species that we identified to possess nsLTPs; (2) Type III is only found in Oryza sativa and Arabidopsis; (3) Types IV and V are shared by Triticum aestivum, Oryza sativa, Sorghum bicolor and Arabidopsis.

**Table 2:** Diversity of the eight cysteine motifs of the nsLTPs recorded in the nsLTPDB

8 CM and number of flanking amino acid residues													
**Type**		**1**		**2**		**3,4**		**5 6**		**7**		**8**	

**I**	X_0-12,16_	**C**	X_8-10_	**C**	X_12-17_	**CC**	X_18-20,29_	**CXC**	X_19-24_	**C**	X_7,13-15_	**C**	X_26,37,48_
**II**	X_0-20_	**C**	X_7_	**C**	X_13,15_	**CC**	X_8-10_	**CXC**	X_16,21,24_	**C**	X_4-7_	**C**	X_0-2_
**III**	X_0-7_	**C**	X_9,10_	**C**	X_12,15,17_	**CC**	X_9_	**CXC**	X_21-24,2_	**C**	X_6-10,13_	**C**	X_0-5,10_
**IV**	X_2-5,10_	**C**	X_14_	**C**	X_14_	**CC**	X_11-13_	**CXC**	X_24_	**C**	X_10_	**C**	X_6,10,12_
**V**	X_2-17_	**C**	X_10_	**C**	X_16,17_	**CC**	X_9_	**CXC**	X_22,23_	**C**	X_7,9_	**C**	X_5-12_

**Cysteine residue numbers are missing**													

**Type**		**1**		**2**		**3,4**		**5 6**		**7**		**8**	

**A**			X_1-13,18,29_	**C**	X_8_,_13-16_	**CC**	X_8,9,16-19_	**CXC**	X_22,23_	**C**	X_6-14_	**C**	X_1-6_
**B**	X_1-3,11_	**C**	X_7,9_	**C**	X_13-16_	**CC**	X_8,12,19_	**CXC**	X_21-24,36_	**C**	X_4,10,13,33,64_		
**C**			X_4,9_	**C**	X_13_	**CC**	X_15,19_	**CXC**	X_22,45_	**C**	X_1,11,12_		
**D**				**C**	X_1-11_	**CC**	X_19_	**CXC**	X_21-23_	**C**	X_4,5,10,12_		

**Table 3:** Molecular weights and pI values of the nsLTPs recorded in the nsLTPDB

Type	Number of species	Number of members	Molecular weight (Mw)	Isoelectric point (pI)
**I**	88	391	7,995-15,000	3.67-12.30
**II**	23	102	6,130-9,270	4.50-12.02
**III**	2	9	7,220-8,918	4.50-6.73, 9.52-10.17
**IV**	4	11	9,348-10,506	8.48-12.31
**V**	3	4	9,282-10,816	4.75-5.27, 9.82

After making the above classification, we further analyzed the pI (isoelectric point) values, Mw (molecular weight) values, charges and the CXC motifs of all available nsLTPs. As shown in Additional File 3A, Type I and Type III were mostly 9 kDa proteins and Type II nsLTPs were 7 kDa proteins; the Mw of Type IV and Type V was much higher than that of Types I-III. Judging from the pI values, Types I, II and III are mostly alkaline proteins. Type IV nsLTPs are weakly alkaline and most Type V nsLTPs are acidic (see Additional File 3B). As for the CXC motif, most residues at the × position in Type I nsLTPs were hydrophilic, while in Type II, III, IV, and V nsLTPs, the × position is usually occupied by a hydrophobic residue (see Additional File 3C). There is no obvious difference in the distribution of net charge among all types of nsLTP (see Additional File 3D).

### Phylogenetic analysis of nsLTPs

In order to analyze the phylogenetic relationships of the nsLTP families, we performed multiple sequence alignments for mature-form nsLTP sequences by using the ClustalW program. Unrooted phylogenetic trees were generated with the UPGMA clustering method implemented in the Phylip package. Based on the number of residues that intervene the eight conserved cysteine residues, the 595 nsLTPs were clustered into 5 different groups. The results of our phylogentic analyses supported our classification results. As shown in Additional File 4 and 5, the 5 types of nsLTPs could be fully separated in the phylogenetic trees.

### Strategies for defining new Prosite-styled patterns for nsLTPs

Functional sites of proteins collected in the Prosite database are expressed as regular expressions. By querying Prosite with the nsLTP sequences that have assigned Uniprot IDs, we noticed that many (*i.e*. 86 cases) of them shared in common the pattern PS00597, that is,

Prosite entry PLANT_LTP: [LIVM]-[PA]-x(2)-C-x-[LIVM]-x-[LIVM]-x-[LIVMFY]-x-[LIVM]-[ST]-x(3)- [DN]-C-x(2)-[LIVM].

**Figure 1:**
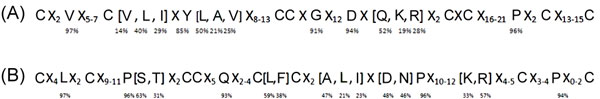
The Prosite-styled pattern for (A) Type I and (B) Type II nsLTPs.

However, this regular expression pattern failed to recognize most of the other nsLTP sequences we collected. Therefore, we would like to define new Prosite-styled patterns that are feasible to identify a broad scope of nsLTPs. We have previously observed, in our multiple sequence alignment results, that several positions in the nsLTP sequence are moderately or even highly conserved. Here we computed the occurrence of 20 amino acids at every position. Notably, in both nsLTP1 and nsLTP2, we found that some positions are always occupied by the same amino acids or amino acids with the same physiochemical properties. Two new patterns for nsLTP Type I and Type II were then defined according to the amino acid occurrence at various positions; they are available in Figure 1. Type III, IV and V were omitted in this experiment because of the small number of cases.

#### The mungbean nsLTP1

**Figure 2:**
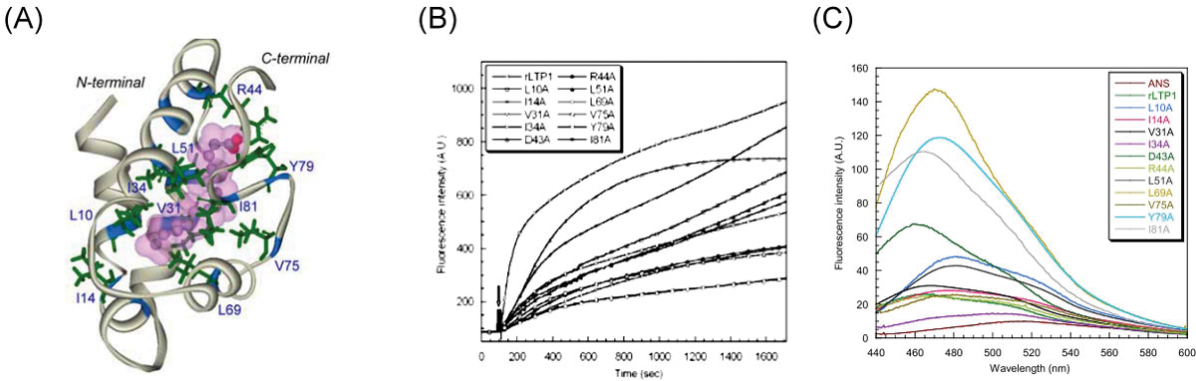
**Structural and functional analyses of mungbean nsLTP1.** (A) CD (Circular dichroism) spectra of the wild-type mungbean nsLTP1 and its mutants. (B) Lipid transfer assay of the wild-type mungbean nsLTP1 and its mutants. (C) ANS (Anilinonaphthalene Sulfonate) binding assay. The mutated sites may interact with lipid molecules.

To evaluate our Prosite-styled pattern for the Type I nsLTPs, we referred to some mutagenesis experimental data and computational results. By using alanine scanning, we have previously identified that Asn9, Leu10, Cys13, Leu17, Leu35, Arg44, Val47, Ala66, Leu69, and Tyr79 are important to the lipid transfer activity of the LTP1 from mungbean because alanine substitutions at these residues increased the lipid transfer activity (Figure 2) [48]. For Leu10, Val31, Ile34, Arg44, Leu51, Leu69 and Val75, which are located in the hydrophobic cavity, this might be because the substituting alanine decreased the hydrophobic stack of the cavity and thus make the structure slightly loosed, creating more space to accommodate the lipid molecules (Figure 2A). Consistently, according to our new Prosite-styled pattern for the Type I nsLTPs, to which the LTP1 of mungbean belongs, the extents of sequence conservation for these functionally important positions were quite high. The occurrence probability for [Leu, Ile or Val] at the position 10, [Ile, Val or Leu] at the position 14, [Val, Ile or Leu] at the position 31, [Ile or Leu] at the position 34, Asp at the position 43, Arg at the position 44, [Leu, Ile, Phe or Val] at the position 51, [Leu or Ile] at the position 69, Val at the position 75, Tyr at the position 79, and Ile at the position 81 are mostly ≥86%. Note that most of the conserved residues are hydrophobic. We supposed that these highly conserved residues may play important roles in nsLTPs.

#### The rice nsLTP2

**Figure 3:**
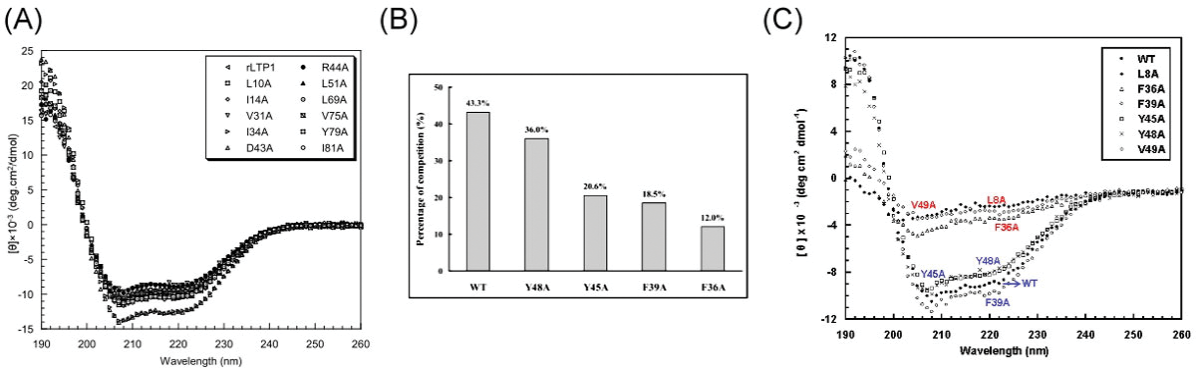
**The structural and functional analyses of rice nsLTP2.** (A) A structure of a rice nsLTP1 myristate complex. (B) Competitive experiment between ANS and LysoPC14. (C) CD spectra of wild-type rice nsLTP2 and its mutants. (B) and (C) were reprinted from our previous data [51].

We have used the protein-protein docking model (Autodock) [49] to investigate the importance of the conserved hydrophobic residues, *e.g*., Leu8, Phe36, Phe39, Tyr45, Tyr48 and Val49, around the binding cavity of rice nsLTP2 (Figure 3A) [50]. The results indicated that changing a single residue of Leu8, Phe36, or Val49 to alanine was sufficient to destroy the integrity of the cavity. Other mutant proteins (*i.e*., F39A, Y45A, and Y48A) typically had native-like structure but were less stabilized compared with the wild type nsLTP2 (Figure 3B and 3C). According to our Prosite-styled pattern for the Type II nsLTPs, to which the rice LTP2 belongs, the sequence of these structurally important residues are highly conserved. The occurrence frequencies for Leu at the position 8, [Phe or Leu] at the position 36, [Phe or Tyr] at the position 39 position, [Tyr or Phe] at position 48, and [Val or Ile] at the position 49 are all ≥92%. The occurrence of [Phe or Leu] at position 45 is also high (75%). Thus, residues with higher conservation may play an important role in structural stability or lipid binding ability.

**Figure 4:**
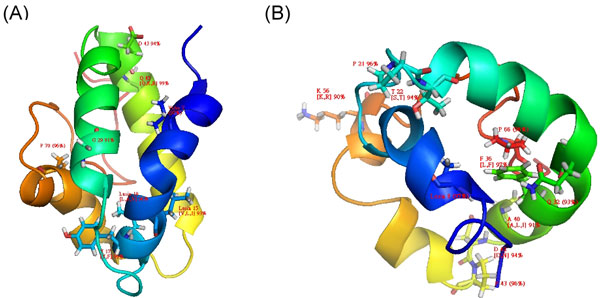
The Prosite-styled patterns of (A) Type I (B) Type II nsLTPs shown in a graphic mode.

These results revealed that our Prosite-styled patterns can provide potential residues that are important to the structural and functional properties of plant nsLTPs. Interestingly, we also noticed that, in the structures of the Type I and Type II nsLTPs, there are several highly conserved (the occurrence frequency of the major amino acids > 90%) positions never studied in previous researches (see Figure 4), and most of these residues are located in alpha-helices or close to the binding cavity for lipids. We supposed that these residues may be good targets for future studies on nsLTPs.

#### The nsLTPDB

**Figure 5:**
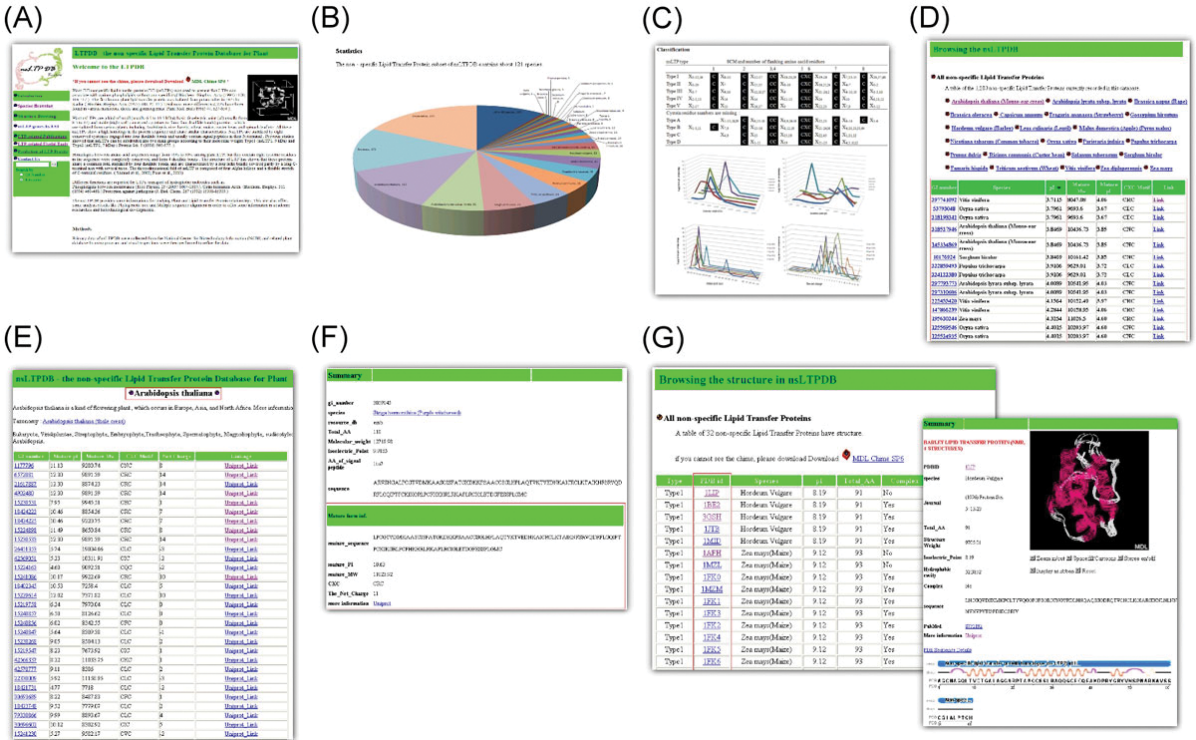
**Page navigation of the nsLTPDB database.** (A) the database homepage, (B) statistics information, (C) classification result, (D) list of collected nsLTPs, (E) the page of species information, (F) the summary of each nsLTP and its mature form information, and (G) the structural summary of each nsLTP.

This database is composed of a web-based user interface collecting all nsLTPs sequence and related information (Figure 5). Our database was made up of five parts including Homepage, Species Browsing, Structure Browsing, Related References, and some useful tools. In our database, there are currently 1,395 putative non-specific lipid transfer protein sequences and 32 PDB structures. Each part is easily accessible by clicking on the hyperlink shown at the left side of the browser window. In addition, the web-based molecular viewer Chem3D http://accelrys.com/products/informatics/cheminformatics/chime/no-fee.php is provided to display the protein conformation. This program allows users to view and manipulate images of molecules structure in three dimensions.

## Conclusions

1. We have constructed an nsLTPs database, which provides the information of sequences, structures, literatures as well as biological data of all plant nsLTPs http://nsltpdb.life.nthu.edu.tw/. There are 595 nsLTPs contained in this database.

2. The phylogenetic tree of the identified nsLTPs was constructed using UPGMA and neighbor-joining clustering algorithms.

3. The 595 nsLTPs were clustered into five different types based on the sequence similarity matrix of them and the properties of their 8-cysteine motifs.

4. We compared the Prosite results with experimental mutagenesis data and found that highly conserved residues in the nsLTP sequence may play an important role in structural stability and/or lipid binding ability of nsLTP.

5. We created Prosite-styled patterns for nsLTPs, which are supposed useful for future identifications and studies of nsLTPs.

## Competing interests

The authors declare that they have no competing interests.

## Authors' contributions

NJW contributed to data collecting, experimental design, data analysis, and database construction. YFY, MNC, and CSC performed wet-lab experiments and analyzed the data. CCL contributed to the design of the web interface of our database and helped analyze computational data. WCL helped review the manuscript. PCL conceived and coordinated the study.

## Supplementary Material

Additional file 1 **The difference between nsLTP1 and nsLTP2.** This file is in PDF format and contains schematic representations of difference between nsLTP1 and nsLTP2. Molecular weight, disulfide bond, 8-Cys patterns, the size of hydrophobic cavity, and the structures are indicated in this file. Click here for file

Additional file 2 **Diversity of the eight cysteine motif in various types of nsLTPs.** This file is in PDF format and contains the diversity of eight cysteine motif of nsLTPs. Click here for file

Additional file 3 **Distribution of (A) Mw, (B) pI, (C) CXC, and (D) net charge of the five types of nsLTPs defined in this work.** This file is in PDF format. Note that in figure (C) × represents a number of intervening residues between two conserved cysteines. Click here for file

Additional file 4 **The unrooted phylogenetic tree constructed with the UPGMA clustering algorithm.** This file is in PDF format and contains phylogenetic tree of 595 nsLTPs. Click here for file

Additional file 5 **The unrooted phylogenetic tree constructed with the neighbor-joining clustering algorithm.** This file is in PDF format and contains phylogenetic tree of 595 nsLTPs. Click here for file
