# Supplementary material for: Construction and analysis of a plant non-specific lipid transfer protein database (nsLTPDB)
Source: BMC Genomics. 2012 Jan 17;13(Suppl 1):S9. doi: 10.1186/1471-2164-13-S1-S9 (PMC3303721; doi:10.1186/1471-2164-13-S1-S9)
Supplement: Additional file 1 — The difference between nsLTP1 and nsLTP2. This file is in PDF format and contains schematic representations of difference between nsLTP1 and nsLTP2. Molecular weight, disulfide bond, 8-Cys patterns, the size of hydrophobic cavity, and the structures are indicated in this file. [file 1471-2164-13-S1-S9-S1.pdf]

The difference between nsLTP1 and nsLTP2.

Type	nsLTP1	nsLTP2
Molecular weight	9 kDa	7 kDa
Disulfide bond	4 disulfide bonds<br>-C [hydrophilic residues] C-	4 disulfide bonds<br>-C [hydrophobic residues] C-
8-Cys patterns	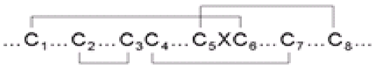	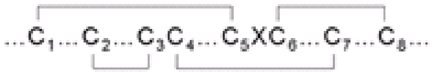
Hydrophobic cavity	Big	Small
Structure	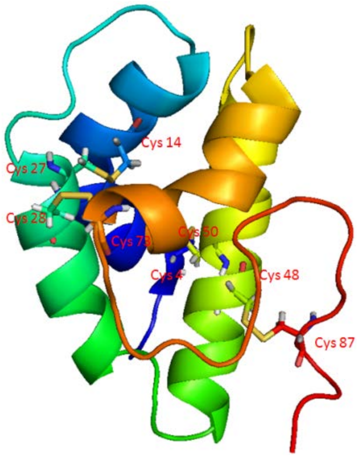	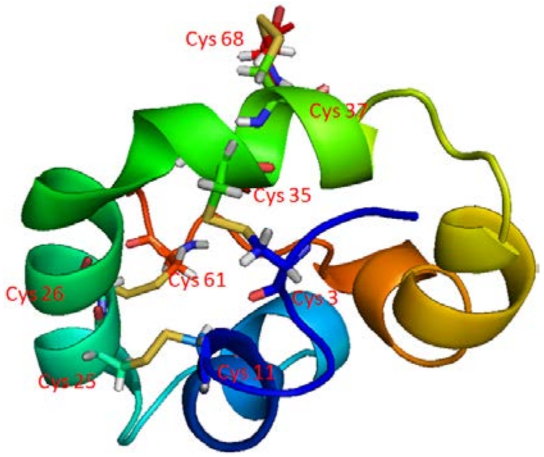
