# Supplementary material for: Construction and analysis of a plant non-specific lipid transfer protein database (nsLTPDB)
Source: BMC Genomics. 2012 Jan 17;13(Suppl 1):S9. doi: 10.1186/1471-2164-13-S1-S9 (PMC3303721; doi:10.1186/1471-2164-13-S1-S9)
Supplement: Additional file 2 — Diversity of the eight cysteine motif in various types of nsLTPs. This file is in PDF format and contains the diversity of eight cysteine motif of nsLTPs. [file 1471-2164-13-S1-S9-S2.pdf]

Diversity of the eight cysteine motif in various types of nsLTPs.

8CM and number of flanking amino acid residues													
Type		1		2		3,4		5 6		7		8	
I	X_{2-9}	C	X_{9}	C	X_{13-15}	CC	X_{19}	CXC	X_{19-24}	C	$X_{7,13,14}$	C	X_{0-26}
II	X_{0-13}	C	X_{7}	C	$X_{13,15}$	CC	X_{8-10}	CXC	$X_{16,21,23}$	C	$X_{5,6}$	C	X_{0-2}
III	X_{2-7}	C	X_{9}	C	$X_{14,16,19}$	CC	X_{9}	CXC	X_{12}	C	X_{6}	C	$X_{1,2,4}$
IV	X_{0-7}	C	$X_{9,10}$	C	X_{15-17}	CC	X_{9}	CXC	$X_{21-24,28}$	C	$X_{6-8,10}$	C	$X_{0,1,5}$
V	$X_{2-5,10}$	C	X_{14}	C	X_{14}	CC	X_{11-13}	CXC	X_{24}	C	X_{10}	C	$X_{6,10,12}$
VI	X_{2-17}	C	X_{10}	C	$X_{16,17}$	CC	X_{9}	CXC	$X_{22,23}$	C	$X_{7,9}$	C	X_{5-12}
VII	$X_{4,50}$	C	X_{9}	C	X_{15}	CC	X_{12}	CXC	X_{27}	C	$X_{9,11}$	C	$X_{17,18}$
VIII	$X_{13,12,21}$	C	X_{6}	C	$X_{13,14}$	CC	X_{12}	CXC	X_{25}	C	X_{8}	C	$X_{2,14,16}$
IX	$X_{2,21}$	C	X_{13}	C	X_{15}	CC	X_{9}	CXC	X_{22}	C	X_{6}	C	$X_{1,4}$

Adopted from the Figure 5 by Gautier, *et al.* in [33]. Species examined are rice, arabidopsis and wheat.

8CM and number of flanking amino acid residues													
Type		1		2		3,4		5 6		7		8	
Ia	$X_{3,4,6,14,15}$	C	X_{9}	C	X_{13-16}	CC	X_{19}	CXC	$X_{22,23}$	C	$X_{10,13,14}$	C	$X_{1,4,7}$
Ib	$X_{2,3,10,12}$	C	X_{9}	C	X_{13}	CC	X_{19}	CXC	X_{22}	C	X_{13}	C	$X_{4,5}$
Ic	X_{3}	C	X_{9}	C	X_{14}	CC	$X_{2}CX_{16}$	CXC	X_{21}	C	X_{13}	C	X_{4}
Id	X_{4}	C	X_{9}	C	$X_{13,16,17}$	CC	X_{19}	CXC	$X_{22,23}$	C	X_{14}	C	$X_{6,15}$
II	X_{2}	C	X_{7}	C	X_{13}	CC	X_{8}	CXC	X_{23}	C	X_{6}	C	X_{0}
IV	X_{3}	C	X_{9}	C	X_{15}	CC	X_{9}	CXC	X_{24}	C	X_{7}	C	X_{0}
IX	X_{2}	C	X_{13}	C	X_{15}	CC	X_{9}	CXC	X_{22}	C	X_{6}	C	X_{4}
X	$X_{2,3,12}$	C	X_{9}	C	$X_{12,14}$	CC	$X_{6,9,20}$	CXC	$X_{22,25}$	C	$X_{13,14}$	C	$X_{4,6,16,17}$

Adopted from the Table 2 by Liu, *et al.* in [34]. Species examined was only Solanaceae.

8CM and number of flanking amino acid residues													
Type		1		2		3,4		5 6		7		8	
1		C	$X_{8,9}$	C	X_{13-17}	CC	X_{18-20}	CXC	X_{19-24}	C	X_{7-14}	C	
2		C	X_{7}	C	$X_{13,15}$	CC	X_{8-10}	CXC	$X_{16,21,23}$	C	X_{5-6}	C	
C		C	X_{9}	C	$X_{14,16,19}$	CC	X_{9}	CXC	X_{12}	C	X_{6}	C	
D		C	$X_{6,14}$	C	X_{8-18}	CC	X_{9-17}	CXC	X_{19-28}	C	X_{6-14}	C	
E		C	$X_{11,13}$	C	X_{15-16}	CC	X_{9}	CXC	X_{21-26}	C	X_{6-7}	C	

F	C	X_{7}	C	X_{11}	CC	X_{10}	CXC	X_{19}	C	X_{9}	C
G	C	X_{6-11}	C	X_{14-19}	CC	X_{12-13}	CXC	X_{20-34}	C	X_{5-12}	C
H	C	$X_{8,11}$	C	$X_{13,16}$	CC	X_{9}	CXC	X_{21}	C	X_{9}	C
J	C	$X_{11,14}$	C	X_{14-15}	CC	X_{12}	CXC	X_{20-22}	C	X_{6}	C
K	C	X_{11}	C	X_{14}	CC	X_{13}	CXC	X_{24}	C	X_{7}	C

Adopted from the Table 1 by Edstam, *et al.* in [53].
