# Supplementary material for: Construction and analysis of a plant non-specific lipid transfer protein database (nsLTPDB)
Source: BMC Genomics. 2012 Jan 17;13(Suppl 1):S9. doi: 10.1186/1471-2164-13-S1-S9 (PMC3303721; doi:10.1186/1471-2164-13-S1-S9)

Distribution of (A) Mw, (B) pI, (C) CXC, and (D) net charge of the five types of nsLTPs defined in this work. Note that in figure (C) X represents a number of intervening residues between two conserved cysteines.

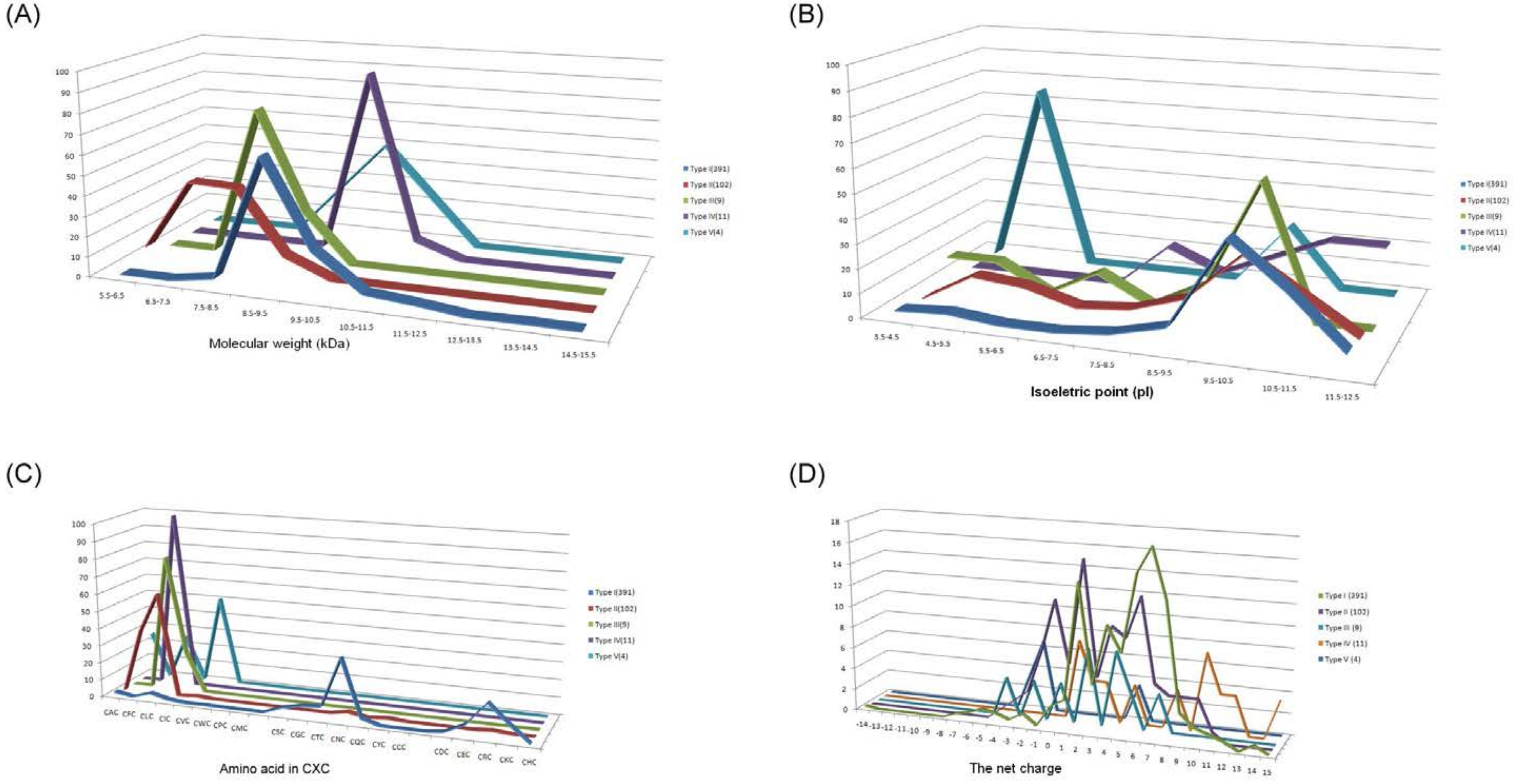

Supplement: Additional file 3 — Distribution of (A) Mw, (B) pI, (C) CXC, and (D) net charge of the five types of nsLTPs defined in this work. This file is in PDF format. Note that in figure (C) × represents a number of intervening residues between two conserved cysteines. [file 1471-2164-13-S1-S9-S3.pdf]
