# Supplementary figures and images for: Construction and analysis of a plant non-specific lipid transfer protein database (nsLTPDB)
Source: BMC Genomics. 2012 Jan 17;13(Suppl 1):S9. doi: 10.1186/1471-2164-13-S1-S9 (PMC3303721; doi:10.1186/1471-2164-13-S1-S9)

**The unrooted phylogenetic tree constructed with the neighbor-joining clustering algorithm.**

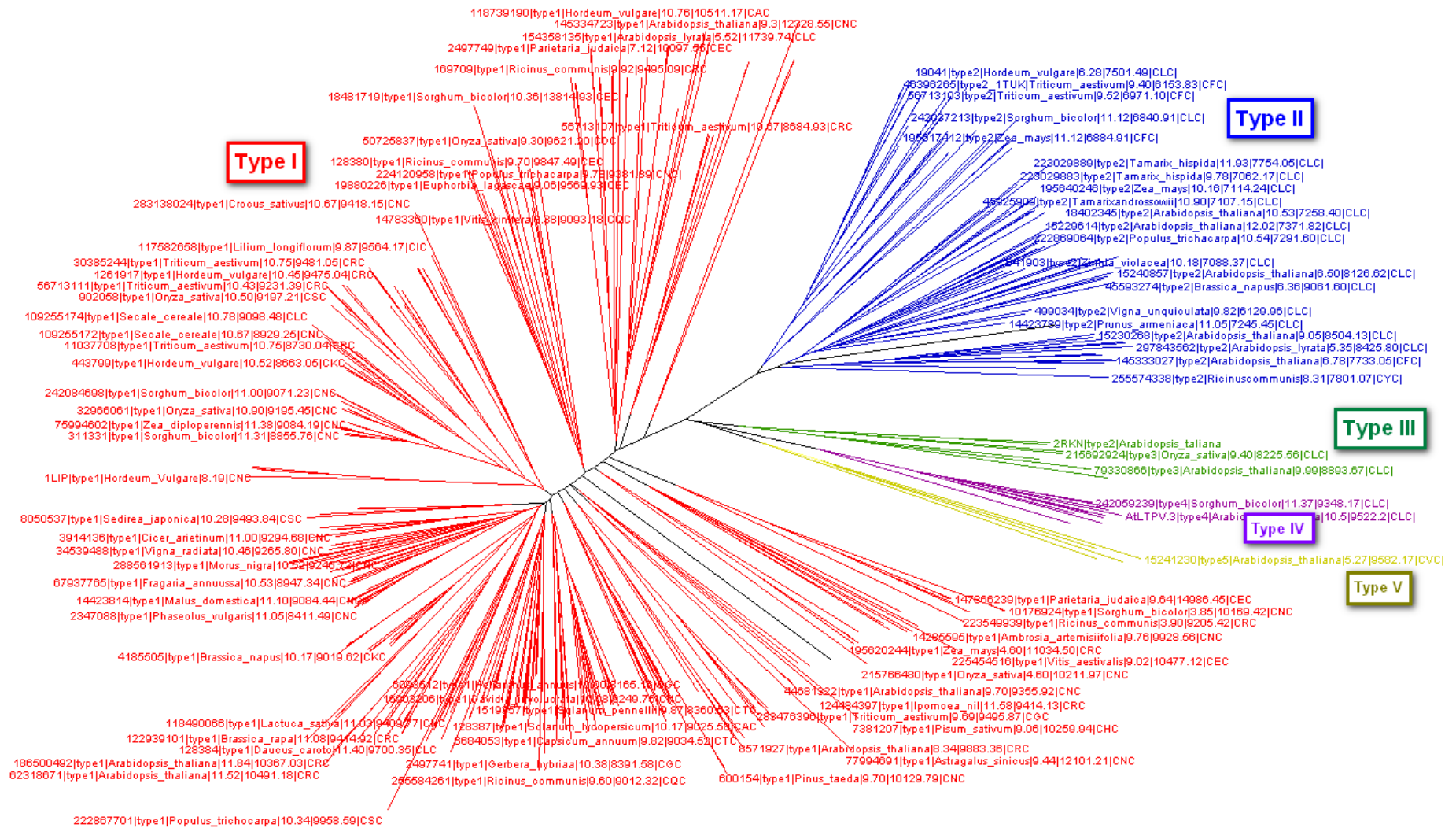

Supplement: Additional file 5 — The unrooted phylogenetic tree constructed with the neighbor-joining clustering algorithm. This file is in PDF format and contains phylogenetic tree of 595 nsLTPs. [file 1471-2164-13-S1-S9-S5.pdf]
